# Early Substance Use Initiation and Suicide Ideation and Attempts among School-Aged Adolescents in Four Pacific Island Countries in Oceania

**DOI:** 10.3390/ijerph121012291

**Published:** 2015-09-30

**Authors:** Karl Peltzer, Supa Pengpid

**Affiliations:** 1 ASEAN Institute for Health Development, Mahidol University, Salaya 73170, Thailand; E-Mail: supaprom@yahoo.com; 2 Department of Research & Innovation, University of Limpopo, Turfloop 0727, South Africa; 3 HIV/AIDS/STIs and TB (HAST), Human Sciences Research Council, Pretoria 0002, South Africa

**Keywords:** early substance use, cigarette smoking, alcohol, drugs, suicidal ideation, suicide attempt, global school-based health survey, Kiribati, Samoa, Solomon Islands, Vanuatu

## Abstract

This study aimed to investigate the correlations between early initiation (<12 years) of smoking cigarettes, alcohol use, and drug use (cannabis) with suicidal ideation and suicide attempts in school-aged adolescents in four Pacific Island countries in Oceania. The sample included 6540 adolescents (≤13 to ≥16 years old) from Kiribati, Samoa, Solomon Islands, and Vanuatu. Bivariate and multivariable analyses were conducted to assess the association between pre-adolescent substance use initiation and suicidal ideation and suicide attempts. Results indicate a prevalence of 25.8% suicidal ideation in the past 12 months (ranging from 17.2% in Vanuatu to 34.7% in Kiribati) and 34.9% suicide attempts in the past 12 months (ranging from 23.5% in Vanuatu to 62.0% in Samoa). The prevalence of early cigarette smoking initiation was 15.7%, early alcohol initiation 13.8%, and early drug use initiation was 12.9%. Students who reported pre-adolescent substance use initiation, compared with non-substance users, were more likely reporting suicidal ideation and suicide attempts. The concurrent initiation of cigarette smoking, alcohol, and drug use should be targeted in early prevention programmes in order to prevent possible subsequent suicidal behaviours.

## 1. Introduction

Young people in many Pacific Island societies, including Samoa, Solomon Islands, and Vanuatu suffer from high rates of suicide and substance use (tobacco use, risky alcohol consumption, high levels of cannabis use) [1,2,3,4,5,6,7,8,9,10,11]. Globally, suicide is the fourth leading cause of death among adolescents [12]. Suicidal behaviour has been defined as suicidal ideation, plans, attempts and also death, through a specific action [13]. Among high school students in five Pacific Island U.S. territories (American Samoa, Commonwealth of the Northern Mariana Islands, Guam, Republic of the Marshall Islands, and Republic of Palau) a median suicidal ideation prevalence of 25.7% and a median prevalence of 19.6% suicide attempts in the past 12 months was found [14]. Other studies among adolescents in the Western Pacific region also found a high prevalence of suicidal ideation, e.g., 17.8% in China [15] and 17.1% in the Philippines [16].

Recent research, particularly in western countries, have shown that early age (pre-teen or preadolescence) of onset of substance use (cigarette smoking, alcohol drinking, cannabis) has been associated with later suicidal behaviours (suicidal ideation and suicide attempts) during adolescence [17,18,19,20,21,22,23]. In a study among adolescents in the U.S. and France, the prevalence of initiating early alcohol use, smoking, and cannabis use were 65.1%, 24.1%, and 3.9%, respectively [22] and in Korea among pre-teens alcohol use initiators the prevalence was 19.9% among boys and 15.2% among girls, and among pre-teen cigarette smoking initiates were 3.2% and 7.9% among boys and girls, respectively [17]. Among high school students in Pacific Island U.S. territories a median of 22.7% had smoked a whole cigarette before age 13, 20.8% drank alcohol before age 13, and 15.4% had tried marijuana for the first time before the age of 13 years [14]. Findings from these studies seem to suggest that early initiation of any substance use may increase the likelihood of suicidal behaviours, while the specific mechanisms for this seem unclear [19]. One possibility is that early substance use is a marker for complex interactions between developmental, psychological, and sociocultural factors that can all contribute to suicidal behaviours among adolescents [19,24,25]. Some studies [16,18] use the problem-behaviour theory of early inititation of substance use and the association with getting involved in multiple health risk behaviours, including suicidal behaviour, among adolescents.

Little is known about early initiation of substance use and suicidal behaviour in developing countries such as in Pacific Island nations. Therefore, this study aims to examine the associations between early initiation (<12 years) of smoking cigarettes, alcohol, and drug use (cannabis) with suicidal ideation and suicide attempts.

## 2. Methods

### 2.1. Description of Survey and Study Population

This study involved secondary analysis of existing data from the Global School-Based Health Survey (GSHS) from four Pacific Island countries in Oceania (Kiribati 2011, Samoa 2011, Solomon Islands 2011, and Vanuatu 2011). All Pacific Island countries from which GSHS datasets with the two-stage cluster sample design and substance use and suicidal behaviour information were publicly available were included in this secondary analysis. Details and data of the GSHS can be accessed [26]. A two-stage cluster sample design was used to collect data to represent all students in grades 6, 7, 8, 9, and 10 in the country. At the first stage of sampling, schools were selected with probability proportional to their reported enrollment size. In the second stage, classes in the selected schools were randomly selected and all students in selected classes were eligible to participate irrespective of their actual ages. Students self-completed the questionnaire under the supervision of trained researchers during classroom periods and recorded responses on a computer scan able answer sheet [26].

### 2.2. Measures

The variables used for this study from the GSHS [26] are described in Table 1. Both of the outcome variables, suicidal ideation in the past 12 months (1 = yes; 0 = no), number of suicide attempts in the past 12 months (1 = any; 0 = none), and potential confounders were dichotomized. Early cigarette smoking, early drinking alcohol, and early drug use initiation were trichotomized into never, prior to 12 years, and 12 or more years.

**Table 1:** Variable description.

Variables	Question	Response Options
**Early cigarette use initiation**	How old were you when you first tried a cigarette?	1 = I have never smoked cigarettes; 2 = 7 years old or younger; 7 = 16 years old or older
**Early alcohol use initiation**	How old were you when you had your first drink of alcohol other than a few sips?	1 = I have never had a drink of alcohol other than a few sips; 2 = 7 years old or younger; 7 = 16 years old or older
**Early drug use initiation**	How old were you when you first used drugs?	1 = I have never used drugs; 2 = 7 years old or younger; 7 = 16 years old or older
**Suicidal ideation**	During the past 12 months, did you make a plan about how you would attempt suicide?	Yes, No
**Suicide attempts**	During the past 12 months, how many times did you actually attempt suicide?	1 = 0 times to 5 = 6 or more times
**Current smoking cigarettes**	During the past 30 days, on how many days did you smoke cigarettes?	1 = 0 days to 7 = All 30 days
**Current alcohol use**	During the past 30 days, on how many days did you have at least one drink containing alcohol?	1 = 0 days to 7 = All 30 days
**Current cannabis use**	During the past 30 days, how many times have you used marijuana?	1 = 0 days to 7 = All 30 days
**Psychological distress**	During the past 12 months, how often have you felt lonely?	1 = never to 5 = always
	How many close friends do you have?	1 = 0 to 4 = 3 or more
	During the past 12 months, how often have you been so worried about something that you could not sleep at night?	1 = never to 5 = always
**Bullied**	During the past 30 days, on how many days were you bullied?	1 = 0 days to 7 = All 30 days
**In physical fight**	During the past 12 months, how many times were you in a physical fight?	1 = 0 times to 8 = 12 or more times

### 2.3. Data Analysis

Data analysis was performed using STATA software version 11.0 (Stata Corporation, College Station, TX, USA). This software has the advantage of directly including robust standard errors that account for the sampling design, *i.e.*, cluster sampling owing to the sampling of school classes. Associations between substance use and suicidal ideation and suicide attempts were evaluated calculating odds ratios (OR). Unconditional logistic regression was used for evaluation of the impact of explanatory variables for suicidal ideation and suicide attempts (binary dependent variables), while controlling for confounding factors. In the analysis, the reported sample size refers to the sample that was asked the target question and weighted percentages are reported. The *P* values less or equal to 5% are used to indicate statistical significance. Both the reported *P* values and 95% confidence intervals are adjusted for the multi-stage stratified cluster sample design of the study.

## 3. Results

### 3.1. Sample Characteristics

The study response rate was for Kiribati 85%, Samoa 79%, Solomon Islands 85%, and Vanuatu 72%. The final sample included 6540 school-going adolescents (51.3% boys and 48.7% girls) predominantly 13–16 years old from Kiribati, Samoa, Solomon Islands and Vanuatu. The study found a prevalence of suicidal ideation was 25.8% (ranging from 17.2% in Vanuatu to 34.7% in Kiribati) and suicide attempt was 34.9% (ranging from 23.5% in Vanuatu to 62.0% in Samoa). Further, the prevalence of early cigarette smoking initiation was 15.7% (19.3% of boys and 10.6% in girls), early alcohol initiation was 13.8% (16.8% of boys and 9.8% in girls), and early drug use initiation was 12.9% (14.9% of boys and 9.5% in girls). Early any substance initiation was 12.2% for one substance, 5.7% for two substances, and 3.4% for three substances. The prevalence of current smoking was 34.2%, current alcohol use 20.6% and current cannabis use 12.2%. Students having one or more psychological distresses (no close friend, mostly or always feeling lonely, or mostly or always being worried/anxiety) was 31.7% (see Table 2).

**Table 2:** Sample characteristics of school-going adolescents in Kiribati, Samoa, Solomon Islands, and Vanuatu.

Variable	Total Sample (*N* = 6540)	Boys (*n* = 2846)	Girls (*n* = 3534)
Age in years			
13 or younger	32.0	31.3	32.7
14	26.0	25.3	27.3
15	24.1	22.7	25.6
16 or older	17.9	20.9	14.4
Suicide ideation: all	25.8	26.8	24.4
Kiribati	34.7	32.8	36.3
Samoa	33.8	37.8	29.5
Solomon Islands	27.7	28.7	25.8
Vanuatu	17.2	18.5	16.1
Suicide attempt: all	34.9	36.7	31.9
Kiribati	31.5	31.5	31.4
Samoa	62.0	68.2	55.7
Solomon Islands	35.0	33.6	34.9
Vanuatu	23.5	28.6	17.7
Cigarette smoking initiation			
Non-initiators	63.1	56.0	72.3
<12 years	15.7	19.3	10.6
≥12 years	21.2	24.8	17.0
Alcohol use initiation			
Non-initiators	67.5	59.2	77.5
<12 years	13.8	16.8	9.8
≥12 years	18.7	24.0	12.7
Drug use initiation			
Non-initiators	74.5	69.1	81.3
<12 years	12.9	14.9	9.5
≥12 years	12.9	16.0	9.1
Substance use initiation <12 years			
Never	78.6	74.0	84.9
One substance	12.2	13.8	10.1
Two substances	5.7	7.6	3.4
Three substances	3.4	4.6	1.5
Current smoking	34.2	40.6	26.5
Current alcohol use	20.6	40.6	14.1
Current cannabis use	12.2	15.6	9.7
Psychological distress			
0	68.3	68.1	68.6
1	26.7	27.1	26.2
2 or 3	5.0	4.8	5.2
Bullied	64.3	65.3	62.2
In physical fight in past 12 months	52.6	57.4	46.6

### 3.2. Association with Suicidal Ideation and Suicide Attempts

Bivariate logistic regression analyses, calculating the associations between substance use initiation and suicidal ideation and suicide attempts are shown in Table 3 and Table 4. Pre-adolescent cigarette smoking initiation, alcohol, and drug use were significantly associated with both suicidal ideation and suicide attempts. Adolescent cigarette smoking initiation, alcohol, and drug use were also significantly associated with both suicidal ideation and suicide attempts. Further, early substance use initiation of one, two, or three substances was positively associated with both suicidal ideation and suicide attempts (see Table 3 and Table 4).

**Table 3:** Proportions and unadjusted Odds Ratios of the study variables by suicidal ideation in school going adolescents in four countries in Oceania.

Variables	Suicidal Ideation					
	Total Sample		Boys		Girls	
	%	OR (95% CI)	%	OR (95% CI)	%	OR (95% CI)
Cigarette smoking initiation						
Non-initiators	17.6	1.00	18.4	1.00	16.9	1.00
<12 years	36.8	2.74 (2.12–3.53) ***	31.5	2.04 (1.4–2.87) ***	47.2	4.41 (2.98–6.52) ***
≥12 years	37.0	2.75 (2.19–3.48) ***	37.0	2.60 (1.9–3.49) ***	36.5	2.84 (2.06–3.90) ***
Alcohol use initiation						
Non-initiators	17.6	1.00	18.4	1.00	17.0	1.00
<12 years	46.9	4.14 (3.52–4.86) ***	44.5	3.55 (2.6–4.74) ***	50.0	4.89 (3.63–6.59) ***
≥12 years	34.6	2.48 (2.01–3.06) ***	32.3	2.12 (1.5–2.92) ***	39.1	3.14 (2.41–4.09) ***
Drug use initiation						
Non-initiators	19.2	1.00	19.5	1.00	18.9	1.00
<12 years	43.7	3.26 (2.57–4.12) ***	44.5	3.31 (2.5–4.27) ***	42.8	3.22 (2.14–4.85) ***
≥12 years	38.4	2.62 (2.09–3.27) ***	35.7	2.29 (1.6–3.27) ***	40.9	2.98 (2.21–4.02) ***
Substance use initiation <12 years						
Never	19.0	1.00	20.3	1.00	17.6	1.00
One substance	29.5	1.78 (1.2–2.62) **	26.5	1.41 (0.4–2.19)	33.1	2.31 (1.23–4.33) **
Two substances	48.4	4.00 (2.9–5.39) ***	44.7	3.17 (2.1–4.72) ***	59.5	6.87 (3.99–11.82) ***
Three substances	44.9	3.46 (2.2–5.31) ***	43.6	3.03 (1.7–5.28) ***	51.5	4.98 (2.38–10.41) ***
Current smoking	44.0	2.73 (2.2–3.31) ***	42.2	2.48 (1.9–3.15) ***	46.4	3.08 (2.40–3.95) ***
Current alcohol use	42.1	2.69 (2.3–3.12) ***	42.1	2.37 (1.9–2.87) ***	45.1	3.20 (2.54–4.03) ***
Current cannabis use	47.8	3.18 (2.5–3.91) ***	45.4	2.76 (2.0–3.64) ***	49.6	3.60 (2.71–4.78) ***
Psychological distress						
0	22.9	1.00	24.1	1.00	21.6	1.00
1	32.0	1.59 (1.25–2.01) ***	31.9	1.48 (1.07–2.05) *	31.8	2.14 (1.43–32.0) ***
2 or 3	37.6	2.03 (1.38–2.99) ***	36.8	1.84 (1.07–3.17) *	37.1	2.14 (1.43–32.0) ***
Bullied	30.5	2.32 (1.97–2.74) ***	32.3	2.53 (1.94–3.30) ***	28.4	2.13 (1.61–2.81) ***
In physical fight in past 12 months	32.0	2.01 (1.75–2.31) ***	32.0	1.77 (1.28–2.43) ***	32.0	2.15 (1.70–2.73) ***

OR = Odds Ratio; CI = Confidence Interval; * *P* < 0.05; ** *P* < 0.01; *** *P* < 0.001.

**Table 4:** Proportions and unadjusted Odds Ratios of the study variables by suicidal attempts in school-going adolescents in four countries in Oceania.

Variables	Suicide Attempts					
	Total Sample		Boys		Girls	
	%	OR (95% CI)	%	OR (95% CI)	%	OR (95% CI)
Cigarette smoking initiation						
Non-initiators	20.2	1.00	20.2	1.00	19.5	1.00
<12 years	59.5	5.81 (4.41–7.65) ***	59.5	5.84 (4.15–8.22) ***	58.4	5.77 (3.75–8.88) ***
≥12 years	48.3	3.69 (2.75–4.95) ***	48.3	3.13 (2.22–4.41) ***	51.3	4.34 (3.26–5.80) ***
Alcohol use initiation						
Non-initiators	19.8	1.00	19.8	1.00	18.9	1.00
<12 years	67.6	8.48 (6.51–11.06) ***	67.6	7.63 (5.18–11.23) ***	70.7	10.36 (7.68–13.97) ***
≥12 years	41.6	2.89 (2.26–3.70) ***	41.6	2.37 (1.75–3.20) ***	36.7	3.75 (2.71–5.19) ***
Drug use initiation						
Non-initiators	22.7	1.00	22.7	1.00	21.9	1.00
<12 years	66.1	6.66 (4.25–10.42) ***	66.1	5.74 (3.26–10.13) ***	67.2	7.30 (4.43–12.02) ***
≥12 years	53.2	3.89 (2.86–5.28) ***	53.3	3.56 (2.45–5.18) ***	55.6	4.46 (3.07–6.49) ***
Substance use initiation <12 years						
Never	20.0	1.00	20.0	1.00	18.8	1.00
One substance	36.5	2.29 (1.58–3.32) ***	36.5	1.73 (0.97–3.11)	39.8	2.84 (1.72–4.69) ***
Two substances	70.1	9.33 (5.69–15.31) ***	70.1	7.25 (4.01–13.12) ***	75.7	13.48 (7.16–21.38) ***
Three substances	75.7	12.44 (7.24–21.38) ***	75.7	10.88 (5.96–19.83) ***	88.6	32.90 (14.07–76.88) ***
Current smoking	62.0	4.29 (3.41–5.41) ***	62.0	4.66 (3.25–6.68) ***	63.4	4.29 (3.39–5.42) ***
Current alcohol use	59.6	4.16 (3.44–5.03) ***	59.6	4.54 (2.85–4.41) ***	62.3	4.98 (3.60–6.88) ***
Current cannabis use	73.3	7.33 (5.47–9.82) ***	73.3	6.72 (4.59–9.83) ***	74.0	8.20 (5.73–11.73) ***
Psychological distress						
0	29.9	1.00	32.4	1.00	26.9	1.00
1	44.3	1.87 (1.47–2.37) ***	45.5	1.75 (1.34–2.34) ***	41.2	1.91 (1.41–2.56) ***
2 or 3	29.9 44.3 59.8	3.49 (2.57–4.74) ***	55.0	2.56 (1.56–4.18) ***	62.7	4.56 (3.23–6.49) ***
Bullied	45.1	4.97 (3.95–6.26) ***	45.1	5.93 (4.51–7.80) ***	41.4	4.13 (3.12–5.48) ***
In physical fight in past 12 months	46.0	3.06 (2.45–3.82) ***	46.0	2.75 (1.96–3.87) ***	44.6	3.14 (2.14–4.09) ***

OR = Odds Ratio; CI = Confidence Interval; * *P* < 0.05; ** *P* < 0.01; *** *P* < 0.001.

Multivariable logistic regression analyses of the associations between the initiation of substance use and suicidal ideation and suicide attempts, adjusted for age and potential confounding variables are shown in Table 5. Pre-adolescent initiation of cigarette smoking was significantly associated with both suicidal ideation and suicide attempts, while adolescent cigarette smoking initiation was neither associated with suicidal ideation nor with suicide attempts. Pre-adolescent and adolescent initiation of cigarette smoking, alcohol, and drug use were associated with both suicidal ideation and suicide attempts. Moreover, the combined early initiation of two or three substances was highly associated with suicide attempts. There were hardly any gender differences, except for a non-association between adolescent alcohol initiation and suicide attempts in boys while there was such an association with girls (see Table 5).

**Table 5:** Adjusted odds ratios for the associations of the initiation of substance use with suicidal ideation and suicide attempts in school-going adolescents in four Oceania countries.

Variables	Suicidal Ideation	Suicide Attempts
	AOR (95% CI)	AOR (95% CI)
**All**		
Smoking ^1^		
-Never	1.00	1.00
-Initiation <12 years	1.65 (1.07–2.53) *	2.12 (1.40–3.22) ***
-Initiation ≥12 years	1.53 (0.96–2.42)	1.21 (0.78–1.89)
Alcohol use ^2^		
-Never	1.00	1.00
-Initiation <12 years	3.39 (2.44–4.71) ***	4.55 (3.34–6.21) ***
-Initiation ≥12 years	1.95 (1.32–2.89) ***	1.64 (1.16–2.32) **
Drug use (illicit) ^3^		
-Never	1.00	1.00
-Initiation <12 years	2.21 (1.48–3.30) ***	2.57 (1.47–4.48) ***
-Initiation ≥12 years	1.88 (1.34–2.63) ***	1.94 (1.29–2.94) **
Substance use initiation <12 years ^4^		
-Never	1.00	1.00
-One	1.49 (1.01–2.16) *	1.39 (0.92–2.12)
-Two	2.11 (1.38–3.24) ***	2.86 (1.79–4.57) ***
-Three	1.70 (0.92–3.14)	3.46 (2.01–5.95) ***
**Boys**		
Smoking ^1^		
-Never	1.00	1.00
-Initiation <12 years	1.04 (0.56–1.94)	1.87 (1.13–2.95) *
-Initiation ≥12 years	1.08 (0.57–2.07)	0.91 (0.52–1.61)
Alcohol use ^2^		
-Never	1.00	1.00
-Initiation <12 years	3.37 (2.16–5.27) ***	3.94 (2.46–6.32) ***
-Initiation ≥12 years	1.88 (1.14–3.10) *	1.19 (0.77–1.85)
Drug use (illicit) ^3^		
-Never	1.00	1.00
-Initiation < 12 years	2.55 (1.70–3.84) ***	2.39 (1.21–4.74) *
-Initiation ≥12 years	1.95 (1.32–2.88) ***	2.07 (1.37–3.13) **
Substance use initiation <12 years ^4^		
-Never	1.00	1.00
-One	1.41 (0.92–2.15)	1.11 (0.65–1.88)
-Three	1.93 (0.86–3.10)	3.31 (1.63–6.73) ***
**Girls**		
Smoking ^1^		
-Never	1.00	1.00
-Initiation <12 years	2.79 (1.64–4.76) ***	2.64 (1.67–4.16) ***
-Initiation ≥12 years	1.83 (1.08–3.10) *	1.56 (0.94–2.58)
Alcohol use ^2^		
-Never	1.00	1.00
-Initiation <12 years	1.90 (1.09–3.70) *	2.87 (1.63–5.07) ***
-Initiation ≥12 years	2.12 (1.34–3.34) **	2.31 (1.51–3.52) ***
Drug use (illicit) ^3^		
-Never	1.00	1.00
-Initiation <12 years	1.90 (1.09–3.70) *	2.87 (1.63–5.07) ***
-Initiation ≥12 years	1.82 (1.04–3.18) *	1.84 (1.05–3.22) *
Substance use initiation <12 years ^4^		
-Never	1.00	1.00
-One	1.59 (0.79–3.17)	1.97 (1.16–3.36) *
-Two	3.16 (1.46–6.83) **	3.35 (1.32–8.46) *
-Three	1.18 (0.52–2.66)	5.44 (1.94–15.25) **

AOR = Adjusted Odds Ratio; CI = Confidence Interval; * *P* < 0.05; ** *P* < 0.01; *** *P* < 0.001; ^1^ Adjusted for age, psychological distress and current smoking; ^2^ Adjusted for age, psychological distress and current alcohol use; ^3^ Adjusted for age, psychological distress and current cannabis use; ^4^ Adjusted for age, psychological distress, current smoking, alcohol, and cannabis use.

## 4. Discussion

The study found considerable rates of pre-adolescent substance use initiation (cigarette smoking, alcohol, and drug use) any very high past year prevalence of suicidal ideation and suicide attempts in four nationally representative samples of school-going adolescents in four Pacific Island countries (Kiribati, Samoa, Solomon Islands, Vanuatu) in Oceania region. Early substance use initiation rates in this study are generally similar to those found in previous studies in five Pacific Island U.S. territories [14], the US [22], Korea [17], and nine developing countries [27]. Suicidal behaviour (ideation and attempts) was much higher in this study as previously found in five Pacific Island U.S. territories [14] and school-going adolescents in Western Pacific countries (China and the Philippines) [16,17]. Several studies have indicated the sharp increase of suicidal behaviour in Pacific Island countries and territories, especially among youth [28,29,30], and also try to explore and hypophesize reasons for this increase in youth suicidal behaviour, including rapid social changes from a more communal village-level of organization to nuclear family socialization of adolescents resulting in intergenerational conflicts, direct and indirect suicide contagion, and co-occurance of mental disorder [28,29]. Previous studies found that young suicide attempters were significantly more likely than non-attempters to have mental health problems such as depression and substance dependence [31]. In this study we measured psychological distress, which was significantly associated with suicidal behaviour. The prevalence of at least one psychological distress was 31.7% in this study, which could be higher than one in eight school students (12–17 years) in Vanuatu who had experienced feelings of servere sadness or depression [32].

In Kiribati, Samoa, Solomon Islands, and Vanuatu pre-adolescent cigarette smoking initiation, alcohol use and drug use initiation were significantly associated with higher prevalences of suicidal ideation and suicide attempts compared to non-smokers, non-drinkers and non-drug users. Initiation of alcohol and drug use, but not cigarette smoking during adolescent years showed similar but less significant associations with suicidal ideation and suicide attempts. Among boys and girls, a similar trend of associations was found in both sexes. These findings confirm previous studies [17,18,19,20,21,22,23] that adolescents who initiate substance use (alcohol, tobacco, and drugs) during pre-adolescence may be at risk to suicidal ideation and suicide attempts in adolescents [17]. Further, the combined early initiation of two or three substances was highly associated with suicide attempts, which was also found among Korean adolescents [17]. These findings show that early substance use and suicidal behaviour is also an important issue in developing or transitional societies such as countries in Oceania [17]. It is possible that adolescents already exposed to pre-adolescent problem behaviours such as substance use may, according to the problem-behaviour theory, engage adolescents in multiple health risk behaviours, including suicidal ideation and suicide attempts [17,18]. Further, research is needed to investigate the underlying patterns of suicidal behaviour. The strongest predictor of pre-adolescence substance among the three substances (cigarette smoking, alcohol, and drug use) for suicide attempts was cigarette smoking. Cho *et al.* [23] also identified a significant positive association between early cigarette smoking initiation, but not alcohol use initiation and suicidal behaviour (ideation) in American adolescents. It is possible that the initiation of cigarette smoking functions more as a gateway drug than other substances such as alcohol use.

## 5. Study Strength and Limitations

A strength of the use of the GSHS was that standardized methods and questionnaires were used across study countries, with nationally representative samples. The study survey was cross-sectional in nature and therefore no causal inferences can be made. Longitudinal studies are required to examine the association between early substance use and suicidal behaviour. Some of the early substance use measures may have limitations, e.g., initiation of cigarette smoking was measured with “when first trying a cigarette”. This less conservative onset variable measurement may have less stronger implications than in some previous studies [19,23], which asked “when you smoked a whole cigarette for the first time,” “started smoking one or more cigarettes / week” or “first got drunk from drinking too much.” Moreover, each variable (e.g., suicidal ideation and suicide attempts) was assessed with a single item instead of using a scale consisting of several items resulting in possible validity and reliability problems. Finally, sadness or depression was not assessed or not available in this publicly available dataset of the GSHS of the study countries. This could have an important comorbity factor for substance use and suicidal behaviour. Instead, a measure of psychological distress (including items of no close friends, loneliness and worry / sleeping problem) was used to remedy this.

## 6. Conclusions

A considerable rate of early substance use (smoking cigarettes, alcohol and drug use) and a high prevalence of suicidal behaviour (ideation and attempts) were found among school-going adolescents in four Pacific Island countries in Oceania. Pre-adolescent initiators of substance use had a higher odds of suicidal behaviours than non-initiators. In addition, more numerous concurrent early substance use behaviours were associated with higher likelihood of suicidal ideation and suicide attempts. Thus, early prevention and intervention activities with concurrent early initiation of cigarette smoking, alcohol, and drug use should be promoted which may in return reduce suicidal behaviours in the future.
